# Practice pattern of physician’s directions of exercise restriction in patients with chronic kidney disease: results from the Chronic Kidney Disease Japan Cohort study

**DOI:** 10.1007/s10157-018-1562-6

**Published:** 2018-03-19

**Authors:** Hiroki Nishiwaki, Takeshi Hasegawa, Megumi Shinji, Fujio Matsuo, Tsuyoshi Watanabe, Hirofumi Makino, Fumihiko Koiwa, Akira Hishida

**Affiliations:** 1 Division of Nephrology, Department of Internal Medicine, Showa University Fujigaoka Hospital, Yokohama, Japan; 2 Center for Innovative Research for Communities and Clinical Excellence, Fukushima Medical University, Fukushima, Japan; 3 Office for Promoting Medical Research, Showa University, 1-5-8 Hatanodai, Shinagawa-ku, Tokyo, 142-8555 Japan; 4 Statcom Company Limited, Tokyo, Japan; 5 Japan Organization of Occupational Health and Safety Fukushima Rosai Hospital, Iwaki, Fukushima Japan; 6 Okayama University, Okayama, Okayama Japan; 7 Yaizu City Hospital, Yaizu, Shizuoka Japan

**Keywords:** Chronic kidney disease, Exercise restrictions, Physical activities, Practice pattern, Adult

## Abstract

**Background:**

The practice patterns of exercise restrictions for patients with chronic kidney disease have not been adequately evaluated yet; thus, we examined them using a cross-sectional design and explored the factors related with those restrictions.

**Methods:**

The Chronic Kidney Disease Japan Cohort study was a multicentre cohort study of Japanese patients (age 20–75 years) living in Japan. We used the information in the questionnaire on the restriction of physical activities offered by physicians to the patients during enrolment. We initially considered and used the following data as the clinical factors that the physician used for decision making on the directions of restriction of physical activities: age, sex, cause of chronic kidney disease (CKD), comorbid diseases, body mass index (BMI), systolic blood pressure, estimated glomerular filtration rate (eGFR) and urine albumin. The logistic regression model was used to explore the factors and estimate their adjusted odds ratio with regard to physician’s direction of restriction of physical activities.

**Results:**

Physician’s direction of exercise restrictions was implemented in 9.9% of the participants. In 17 facilities, the proportion of physician’s direction of exercise restriction ranged from 2.9 to 17.8%. The logistic regression analysis showed that the proportion of the factors such as younger age, cardiovascular disease, congestive heart failure and lower eGFR was higher in patients with physician’s direction of exercise restrictions.

**Conclusions:**

The findings from this study suggested the factors related with prescribing exercise restrictions. Further studies examining which patients with CKD need direction of exercise restrictions are needed.

## Introduction

Exercise has been recommended for the general population because of the evidence of an inverse dose–response relationship between physical activity and all-cause mortality and morbidity in multiple patient populations including those with cardiovascular disease (CVD), thromboembolic stroke, hypertension, osteoporotic fractures, obesity, type 2 diabetes mellitus, colon and breast cancer, anxiety and depression [1].

Post-exercise proteinuria is frequent in healthy subjects [2]. However, the modifications induced by physical exercise on urinary protein excretion and worsening renal dysfunction in human subjects with proteinuric nephropathies were not evaluated. Some nephrologist have thought that exercise, by increasing proteinuria, might have dangerous effects on the renal function of patients with chronic kidney disease (CKD) [3]. Otherwise, some randomised control trials (RCTs) on exercise for CKD were conducted [4–6]. The results showed no change in GFR decline between the groups. The results were unclear if they were accurate or if they were due to the short study duration, low statistical power or intensity of exercise. There are several prospective studies examining the relationship between exercise and CKD. A prospective non-RCT study showed that patients with CKD in an exercise group did not start dialysis or die in 10 years, i.e., they did not reach the study endpoint (renal replacement therapy or all-cause death), whereas more than half of the control group reached the study endpoint [7].

The Kidney Disease: Improving Global Outcomes guideline recommends that people with CKD undertake physical activities that are compatible with cardiovascular health and tolerance (aiming for at least 30 min, 5 times per week) [8]. Meanwhile, the Japanese guideline for patients with CKD did not definitively recommend exercises for patients with CKD [9].

As seen above, the findings regarding exercise and renal function in patients with CKD have been accumulated; the results indicated that exercise was beneficial, or at least not harmful, for patients with CKD; however, there seems to be an evidence–practice gap regarding exercise for CKD patients [3]. To our knowledge, the recent practice pattern of physician’s direction of exercise restriction for patient with CKD has not been evaluated. Our hypothesis was that physicians could have imposed unnecessary exercise restrictions on patients with CKD, regardless of recent findings. In the present study, we examined the practice patterns of physician’s direction of exercise restrictions in patients with CKD and explored the factors related to those restrictions to clarify the real-world situation of these patients.

## Materials and methods

### Design

We used a cross-sectional design to determine the factors related to restriction of physical activities.

### Setting and participants

The Chronic Kidney Disease Japan Cohort (CKD-JAC) study was multicentre cohort study, conducted from April 2007 to March 2013, of Japanese patients living in Japan who have CKD (age 20–75 years; eGFR 10–59 mL/min/1.73 m^2^) [10, 11]. The following were excluded from the CKD-JAC study: patients with polycystic kidney disease, human immunodeficiency virus infection, liver cirrhosis, active cancer or cancer treatment within last 2 years; transplant recipients and patients who have previously received long-term dialysis; individuals who refused to provide informed consent; those without data on age, sex, outcome measure, serum creatinine at enrolment and information on restriction of physical activities.

### Variables, data sources and measurements

We used the enrolment data and the information in the questionnaire on physician’s direction on restriction of physical activities offered by the physician at enrolment (to which the patients replied with either ‘yes’ or ‘no’). The questionnaire asked “Have you received a directive by your physician to restrict exercise?” We initially used the following as clinical factors related to restriction of physical activities: age, sex, cause of CKD, comorbid diseases [such as CVD, congestive heart failure (CHF) and stroke], body mass index (BMI), systolic blood pressure, eGFR and urine albumin. We also assessed the CKD stages to confirm the nonlinear association between eGFR and exercise restrictions. To evaluate poorly controlled hypertension, we defined uncontrollable hypertension as systolic blood pressure > 180 mmHg and/or diastolic pressure of 110 mmHg. In the final model, we used the variables age, sex, cause of CKD, comorbid disease (CVD, CHF and stroke), BMI, systolic blood pressure, eGFR and urine albumin because only few patients had uncontrollable hypertension and eGFR has a linear relationship with exercise restrictions. The measurements of each variable were described elsewhere [10].

### Statistical methods

We described the patient characteristics based on physician’s direction of restriction of physical activities (no restriction or restriction). Continuous data were summarised as mean values (standard deviation) and median (first and third quartiles), and categorical data as proportions. Comparisons of continuous variables among the three groups were assessed using the analysis of variance (ANOVA), and comparisons of categorical data were assessed using the Chi-square test. The logistic regression model was used to explore the factors and estimate the adjusted relative risk of the factors with regard to physician’s direction of restriction of physical activities. Considering the differences in practice patterns regarding the physician’s directive to restrict physical activities, we also performed a conditional logistic regression analysis with each facility as a stratum. To examine the frequency of the direction of exercise restriction by the facilities, we divided all 17 facilities into three groups by the frequency of the direction of exercise restriction. We defined the lowest frequency of the direction of exercise restriction as the 1st tertile and the highest as the 3rd tertile. We described the patient characteristics by each groups. We performed a complete case analysis. All analyses were conducted using SAS software, version 9.4 (SAS Institute, Cary, NC, USA), with two-sided significance set at 0.05.

## Results

### Participant characteristics

Of the 2966 eligible enrolled patients, only 2565 patients, whose information on physician’s direction of restriction of physical activities was documented at study initiation, were analysed, since 401 subjects did not have such information (Table 1).

**Table 1:** Patient characteristics by exercise restrictions

	Exercise restrictions		All
	No	Yes	
	*n* = 2312 (90.1%)	*n* = 253 (9.9%)	*n* = 2565 (92.9%)
Age, years (SD)	60.6 (11.4)	57.6 (12.8)	60.3 (11.6)
Gender, female, %	37.6%	39.5%	37.8%
Cause of CKD, *n* (%)			
Diabetic nephropathy	427 (18.5%)	62 (24.5%)	289 (19.1%)
Non-diabetic	1885 (81.5%)	191 (75.5%)	2076 (80.9%)
Comorbidities			
Cardiovascular disease, %	12.8%	17.4%	13.3%
Stroke, %	9.8%	8.7%	9.7%
Congestive heart failure, %	3.4%	6.3%	3.7%
Smoking			
Active smoker	17.1%	15.1%	16.9%
Ex-smoker	27.9%	27.8%	27.9%
Non-smoker	55.0%	57.1%	55.2%
Alcohol use	39.8%	34.3%	39.2%

Exercise Restrictions: We used the information about the restriction of the physical activities offered by the physician in the questionnaire at the enrollment. The patients reported “Yes” or “No” about the restriction of the exercise in this questionnaire

*SD* standard deviation, *CKD* Chronic Kidney disease

### Exercise restriction pattern

**Fig. 1:**
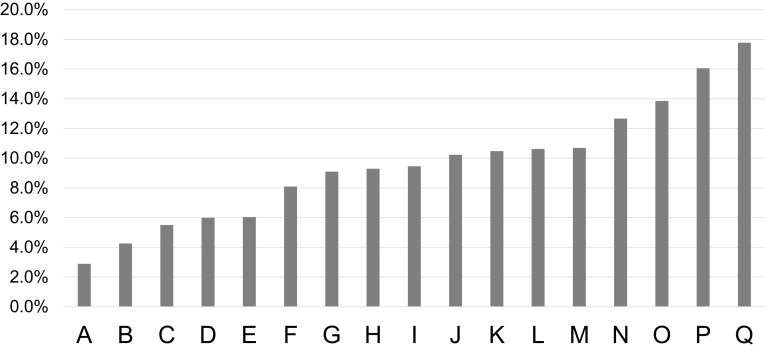
Rate of the exercise restrictions by the facilities. Facility A–E: 1st tertile, F–K: 2nd tertile, L–Q: 3rd tertile

We showed the distribution of the practice of physician’s direction of restriction exercise (Table 2). In 17 facilities, the proportion of physician’s direction of restriction exercise ranged from 2.9 to 17.8% (Fig. 1). Table 3 shows the patient’s characteristics by tertile of proportion of physician’s direction of exercise restriction. Statistical significance and dose–response relationship were found in total cholesterol and urine albumin.

**Table 2:** Laboratory data by exercise restrictions

	Exercise restrictions		All
	No	Yes	
	*n* = 2312 (90.1%)	*n* = 253 (9.9%)	*n* = 2565 (92.9%)
BMI (SD)	23.5 (3.78)	23.2 (4.1)	23.5 (3.8)
Systolic blood pressure, mmHg (SD)	132 (18)	132 (18)	132 (18)
Diastolic blood pressure, mmHg (SD)	77 (12)	76 (12)	77 (12)
Uncontrollable hypertension, *n* (%)	32 (1.4%)	1 (0.4%)	33 (1.3%)
Hemoglobin, g/dl (SD)	12.1 (1.9)	11.7 (1.7)	12.1 (1.9)
Hematocrit, % (SD)	36.4 (5.3)	35.1 (4.9)	36.2 (5.3)
Serum creatinine, mg/dl (SD)	2.1 (1.1)	2.3 (1.2)	2.1 (1.1)
eGFR, ml/min/1.73 m^2^ (SD)	29.1 (12.4)	27.3 (11.8)	28.9 (12.3)
CKD stage, *n* (%)			
3a	231 (10.6%)	19 (8.3%)	250 (10.4%)
3b	765 (35.0%)	69 (30.0%)	834 (34.5%)
4	837 (38.3%)	102 (44.3%)	939 (38.9%)
5	339 (15.5%)	40 (17.4%)	379 (15.7%)
Urine albumin, mg/g Cr (SD)	941.2 (1252.5)	1113.1 (1515.1)	957.6 (1280.3)

Uncontrollable hypertension was defined as more than 180 mmHg in the systolic blood pressure and/or 110 mmHg in diastolic pressure

*BMI* body mass index, *SD* standard deviation, *eGFR* estimated glomerular filtration rate, *CKD* chronic kidney disease

**Table 3:** Patient’s characteristics by tertile of proportion of physician’s direction of exercise restriction

	Tertile of proportion of exercise restriction each facilities			*p* value
	1st tertile	2nd tertile	3rd tertile	
	*n* = 696	*n* = 880	*n* = 989	
Age	61.2 (10.7)	59.9 (12.0)	59.9 (11.7)	0.44*
Gender (female)	284 (40.8%)	309 (35.1%)	377 (38.1%)	0.62
Diabetic nephropathy	123 (17.7%)	157 (17.8%)	209 (21.1%)	0.11
CVD	118 (17.0%)	99 (11.3%)	123 (12.4%)	< 0.01*☨
Stroke	61 (8.8%)	82 (9.3%)	105 (10.6%)	0.41
CHF	24.0 (3.4%)	25 (2.8%)	45 (4.6%)	0.14
BMI	23.5 (3.78)	23.25 (3.74)	23.66 (3.89)	0.08*
Systolic blood pressure	134.3 (19.1)	127.9 (17.8)	133.4 (17.8)	< 0.01*^☨^
Uncontrollable hypertension	13 (1.9%)	8 (0.9%)	12 (1.2%)	0.21
eGFR	8 (12.27)	29.38 (12.35)	29.09 (12.34)	0.10
CKD stage				0.54
3a	70 (10.3%)	88 (10.4%)	92 (10.4%)	
3b	219 (32.2%)	297 (35.1%)	318 (35.9%)	
4	269.0 (39.5%)	337 (39.8%)	333 (37.6%)	
5	121 (17.8%)	119 (14.0%)	139 (15.7%)	
Albuminuria	834 (1114.29)	951.52 (1322.45)	1059.05 (1350.34)	< 0.01*^☨^
Total cholesterol	188.1 (37.8)	193.9 (47.2)	197.9 (41.2)	< 0.01*^☨^

Uncontrollable hypertension was defined as more than 180 mmHg in the systolic blood pressure and/or 110 mmHg in diastolic pressure

*BMI* body mass index, *CVD* cardiovascular disease, *CHF* congestive heart failure, *eGFR* estimated glomerular filtration rate, *CKD* chronic kidney disease

*< 0.05, Comparisons were assessed using the chi-squire test, except for ☨ using ANOVA

### Factors associated with exercise restrictions

We showed the univariable and multivariable logistic regression analyses for physician’s direction on restriction of physical activities (Table 4). In the univariable analysis, age (10-year increments), diabetic nephropathy, CVD, CHF and eGFR were related to physician’s direction of restriction of physical activities. In the multiple variable analysis, age (10-year increments), CVD, CHF and eGFR were related to the outcome. The analysis of variables stratified by facilities showed a similar trend.

**Table 4:** Logistic Analysis for the Exercise Restrictions and Data Missing

	Univariate				Multivariate				Data missing (*n*, %)	
	Not stratified		Stratified by the facilities		Not stratified		Stratified by the facilities			
	OR		OR		OR		OR			
	Ratio	95% CI	Ratio	95% CI	Ratio	95% CI	Ratio	95% CI		
Age^a^	0.81*	0.73–0.90	0.81*	0.73–0.90	0.75*	0.66–0.86	0.75*	0.66–0.86	0	0.0
Gender	0.92	0.71–1.20	0.91	0.69–1.19	0.84	0.62–1.14	0.83	0.61–1.13	0	0.0
Diabetic nephropathy	1.43*	1.06–1.94	1.45*	1.06–1.99	1.45	1.00–2.11	1.44	0.98–2.12	0	0.0
CVD	1.43*	1.01–2.03	1.58*	1.10–2.25	1.66*	1.11–2.50	1.85*	1.22–2.82	0	0.0
Stroke	0.88	0.56–1.39	0.9	0.54–1.37	0.94	0.56–1.59	0.92	0.54–1.57	0	0.0
CHF	1.93*	1.11–3.37	1.83*	1.04–3.23	2.21*	1.23–3.97	2.07*	1.14–3.78	0	0.0
BMI	0.98	0.94–1.01	0.98	0.94–1.01	0.97	0.93–1.01	0.97	0.93–1.01	239	9.3
Systolic blood pressure	1.00	0.93–1.07	1.00	0.92–1.07	1.00	0.93–1.11	1.02	0.94–1.12	25	1.0
Uncontrollable hypertension	0.28	0.04–2.06	0.30	0.04–2.22					60	2.3
eGFR^a^	0.89*	0.79–0.99	0.88*	0.78–0.98	0.87*	0.77–0.99	0.86*	0.75–0.97	151	5.9
CKD stage										
3a	Reference		Reference							
3b	1.10	0.65–1.86	1.09	0.64–1.86						
4	1.48	0.89–2.47	1.52	0.91–2.54						
5	1.43	0.81–2.54	1.48	0.83–2.63						
Urine albumin^b^	1.05	1.00–1.10	1.04	0.99–1.09	1.00	0.94–1.06	0.98	0.93–1.05	187	7.3

“Stratified by the facilities” is the logistic regression analysis stratified by the facilities

*OR* odds ratio, *CKD* chronic kidney disease, *CVD* cardiovascular disease, *CHF* congestive heart failure, *BMI* body mass index, *eGFR* estimated glomerular filtration rate

*p < 0.05

^a^ Unit by 10

^b^ Unit by 500

## Discussions

In the present study, 9.9% participants were given physician’s direction of exercise restrictions. The logistic regression analysis showed that younger age, CVD, CHF and lower eGFR are related to prescribing exercise restrictions. On the contrary, sex, cause of CKD (diabetic or not), stroke, BMI, systolic blood pressure and albuminuria were not related with it.

The present study showed the variations between institutions in terms of physician’s directions about exercise restriction. As mentioned in the introduction, many studies [5–7] reported that prescribing exercise for patients with CKD has good effects on the change in eGFR and proteinuria, exercise capacity and mortality. In a Japanese guideline, the recommendation for exercise was not clearly stated, but it recommended that physicians should make plans about exercise, considering the activity, exercise capacity and risk of CVD.

We noted that the younger the participants, the more they were inclined to be given physician’s direction of exercise restrictions. As younger participants might exercise more than older ones, physicians might be imposing exercise restrictions for younger patients with CKD [12, 13]. History of CVD or CHF was associated with physician’s direction of exercise restrictions. The American College of Cardiology Foundation/American Heart Association (AHA) recommended that exercise training (or regular physical activity) is recommended as safe and effective for patients with heart failure who can participate in the improvement of functional status [14]. Thus, those physicians’ direction of exercise restrictions could be inappropriate. The lower the eGFR, the more the patients were given physician’s direction of exercise restrictions. According to Robinson-Cohen et al. [15], there was little difference in the degree of eGFR change in the sub-analysis based on CKD stages, regardless of the amount of physical activities.

Diabetic nephropathy as cause of CKD was not associated with physician’s direction of exercise restriction in this study. We predicted the opposite results, that is, diabetic nephropathy was associated with physician’s direction of exercise restrictions because diabetic neuropathy was expected in patients with diabetic nephropathy [16]. The present study showed marginal results. The almost similar ORs and 95% CIs obtained from logistic regression analyses of variables that were and were not stratified by facilities could have resulted from a lack of sample. Moderate to severe stroke generally causes disability, and the AHA/American Stroke Association recommended physical exercise to reduce stroke factors [17]. In the present study, the history of stroke was not associated with physician’s direction of exercise restrictions. As in the recommendation, exercise should be actively recommended for patients with stroke; however, there is a possibility that physician’s direction of exercise restrictions was not prescribed in patients with decreased activities of daily living due to the presence of disability. As some authors recommend, patients with uncontrolled high blood pressure should avoid exercising [18]. In the present study, there were few patients with uncontrolled hypertension; thus, we could not show any relationship between uncontrolled hypertension and physician’s direction of exercise restrictions. There was also no relationship between systolic blood pressure as continuous variable and physician’s direction of exercise restrictions or between BMI and physician’s direction of exercise restrictions. A pilot trial in patients with obesity, type 2 diabetes mellitus and CKD showed little difference in proteinuria and eGFR [6]. However, there was some evidence that weight loss intervention in CKD is associated with decreasing proteinuria with no further decrease in GFR [19, 20]. Exercise along with weight loss might be beneficial for obese patients with CKD.

Our findings showed that albuminuria was not associated with physician’s direction of exercise restrictions, despite a report that exercise can increase proteinuria with abnormal glomerular permeability [2]. Nephrologists are concerned that it might accelerate the progression of renal dysfunction [3], but recent studies showed that physical activities are not associated with progression of renal disease [5–7]. However, in facilities that prescribed the more exercise restrictions, patients were likely to have more albuminuria (Table 3). There was no association between albuminuria and restriction of exercise in the analysis for each individual; however, there was a relationship between albuminuria and the frequency of the directions of exercise restrictions by facilities. These results might suggest that the practice pattern or the attitude towards exercise for patients with CKD of each facility affect physician’s direction of exercise restriction, that is, as mentioned in the introduction, nephrologists used to consider proteinuria through increasing glomerular permeability from exercise as the cause of CKD progression [3]. However, many recent clinical studies revealed opposite results [5–7]. The variation in practice pattern might be caused by the facility’s policy based on the old concept, and we might just look at the transition time of the practice pattern of exercise therapy for the patients with CKD or the evidence–practice gap [21]. In facilities that prescribed more exercise restrictions, patients were likely to have higher total cholesterol; however, the difference in the absolute value of total cholesterol between the 1st tertile and the 3rd tertile was not clinically significant.

This study has several strengths. First, the CKD-JAC study has enrolled a cohort of individuals with CKD who were examined by nephrologists; thus, major Japanese facilities took part in this cohort. Second, this study was one of the very few studies that examined the practice pattern of physician’s direction of exercise restrictions for patients with CKD. As stated earlier, some of the findings of this study suggested the presence of evidence–practice gap; thus, we expect that this study would help improve the quality of care and lead to more studies on exercises for patients with CKD.

However, several limitations should also be mentioned. First, the population in this study was limited to Japanese patients, hampering generalisability to a global population. Second, the variable of physician’s direction of exercise restrictions by physicians was binary, with patients reporting the variable in the questionnaire, which can possibly lead to incorrect estimation of prevalence of physician’s direction of exercise restrictions, and the details of the exercise restrictions, such as intensity, length and frequency, were unclear. We recommended that studies with detailed information about these exercise restrictions be performed in the future. Third, we considered that not only the patient characteristics but also the facility characteristics affected the practice pattern. For example, the availability of consultation for rehabilitation medicine or cardiology services can influence the proportion of exercise restriction. All 17 facilities have a department of rehabilitation medicine and a cardiology department. Therefore, our results could have only limited application for a general hospital. Fourth, some data, such as eGFR, were missing. Our analysis did not include 5.9% of patients whose eGFR was unknown. The variable of eGFR for inclusion criteria was measured in each facility. Otherwise, the variable of eGFR used in the baseline data was measured at the central laboratory. This is why the eGFR level was one of the inclusion criteria of eGFR but there were some patients without eGFR at baseline. These missing data could have some impact on the results. Finally, because the status of diabetes mellitus, CVD and CHF was unclear, we could not examine the influence of diabetic retinopathy, diabetic neuropathy and instability of CVD and CHF on exercise restrictions.

## Conclusion

In conclusion, 9.9% of patients with CKD were given physician’s direction of exercise restrictions, and the factors, younger age, CVD, CHF and lower eGFR, are associated with exercise restrictions. Exercise is important for the prevention of CVD. Thus, further studies that examine the effect of exercise on the progression of CKD and the incidence of CVD in younger patients with CKD, patients with CVD/CHF and CKD and patients with lower eGFR are needed. We expect that this study would help improve the quality of care and lead to further studies on exercises for patients with CKD.
